# Extremely Sparse Olfactory Inputs Are Sufficient to Mediate Innate Aversion in *Drosophila*

**DOI:** 10.1371/journal.pone.0125986

**Published:** 2015-04-30

**Authors:** Xiaojing J. Gao, Thomas R. Clandinin, Liqun Luo

**Affiliations:** 1 Howard Hughes Medical Institute and Department of Biology, Stanford University, Stanford, California, United States of America; 2 Department of Neurobiology, Stanford University, Stanford, California, United States of America; Center for Genomic Regulation, SPAIN

## Abstract

Innate attraction and aversion to odorants are observed throughout the animal kingdom, but how olfactory circuits encode such valences is not well understood, despite extensive anatomical and functional knowledge. In *Drosophila melanogaster*, ~50 types of olfactory receptor neurons (ORNs) each express a unique receptor gene, and relay information to a cognate type of projection neurons (PNs). To examine the extent to which the population activity of ORNs is required for olfactory behavior, we developed a genetic strategy to block all ORN outputs, and then to restore output in specific types. Unlike attraction, aversion was unaffected by simultaneous silencing of many ORNs, and even single ORN types previously shown to convey neutral valence sufficed to mediate aversion. Thus, aversion may rely on specific activity patterns in individual ORNs rather than the number or identity of activated ORNs. ORN activity is relayed into the brain by downstream circuits, with excitatory PNs (ePN) representing a major output. We found that silencing the majority of ePNs did not affect aversion, even when ePNs directly downstream of single restored ORN types were silenced. Our data demonstrate the robustness of olfactory aversion, and suggest that its circuit mechanism is qualitatively different from attraction.

## Introduction

Olfactory circuits in insects and mammals exhibit striking functional and anatomical similarities [1]. Functionally, olfactory inputs are high dimensional, corresponding to the activation patterns of ~50 types of olfactory receptor neurons (ORNs) in flies and ~1000 ORN types in mice. Among these input channels, a minority are “specialists”, responding specifically to one odorant or one category of odorants [2–4]. Most input channels, however, are “generalists” where one odorant activates multiple types of ORNs and one ORN type responds to multiple odorants [5]. Anatomically, ORNs of the same type express the same receptor, and project to the same glomerulus in the antennal lobes in flies [6–8] or olfactory bulbs in mice [9, 10]; each projection neuron (PN) in flies or mitral/tufted cell in mice then relays information from a single glomerulus to higher brain centers for associative learning and innate behavior [11–14]. Such similarities in organization across diverse phyla suggest that convergent evolution might have produced a particular neural anatomy that suits olfactory processing.

What aspects of ORN activity are required for higher brain centers to direct olfactory aversion and attraction [15]? In *C*. *elegans* each sensory neuron is preferentially linked to aversion or attraction [16, 17]. However, unlike insects and mammals, worms have much fewer olfactory sensory neurons, each of which expresses multiple receptor genes [18]. It is thus unclear whether the conclusions from worms are applicable to olfactory systems with more neurons and neuronal types. Here, we exploit the genetic tools in *Drosophila melanogaster* to probe the causal relationship between ORN activity and innate olfactory aversion.

Similar to *C*. *elegans*, aversion can be induced in flies when single “specialist” ORN types are activated by specific repellents, such as CO_2_ [3] or geosmin [4], emitted by other stressed flies or toxic microbes, respectively. The corresponding ORNs are thus believed to stereotypically convey aversion. However, how “generalist” ORN activities are relevant for aversion remains controversial. Flies tend to avoid odorants at higher concentrations, regardless of chemical compositions [19], and increased odorant concentration activates more ORN types [5]. Three classes of hypotheses are consistent with these observations, the conceptual elaborations of which can be found in a recent review on intensity coding in mammalian olfaction [20]. First, for aversion, downstream circuits may extract a population metric such as the number of activated ORN classes (Hypothesis I). Alternately, a downstream aversion circuit may be hardwired to ORNs expressing specific receptors. In this model, aversion-specific ORNs with low and non-specific odorant affinity may signal only when any odorant reaches sufficiently high concentrations (Hypothesis II). In a third scenario, specific activity patterns within individual ORNs may be interpreted by higher brain centers as signals of aversive cues, while the number or identity of activated ORNs is incidental. For example, as odorant concentration increases, aversion could be encoded by elevated firing rate or reduced latency in any “generalist” ORNs.

The identity of activated ORNs have been correlated with aversion and attraction in larval [21] and adult flies [22]. However, these studies implicitly adopted Hypothesis II and could not causally test other possibilities. Meanwhile, attempts to establish causal relations were limited by the fact that ORNs can be divided into two major classes expressing either odorant receptor (OR) family [23] or ionotropic receptor (IR) family of receptors [24]. Previous studies only manipulated either OR+ or IR+ ORNs, while outputs from the other class complicated the interpretation of results [25, 26]. To overcome these limitations, we applied a genetic strategy of blocking all ORN activity, and then restoring activity in ORNs expressing specific receptors. Our results indicate that aversion does not require the overall ORN activity pattern, and that a single ORN type can convey aversion. Moreover, our data demonstrate that aversion is intact after inactivating a large subset of ePNs that express *GH146-GAL4*, including those directly post-synaptic to the active ORNs, long considered to be the major output route from the antennal lobe. Our data constrain future models of valence coding in the olfactory circuit.

## Material and Methods

### Fly Stocks

The following flies were used: *UAS-shi* ^*ts1*^ [27], *Orco-GAL4* [23], *Ir8a-GAL4* [28], *Pebbled-GAL4 [29], ey-FLP* [30], *UAS-FRT-stop-RTF-shi* ^*ts1*^ (II, III) [31], *UAS-FRT-stop-FRT-CD8*::*GFP* [32], *UAS-nsyb*::*GFP* [33], *Orco-GAL80* [34], *Ir64a-GAL80* [26], *GH146-GAL4* [35], *GH146-FLP* [32], *GH146-QF* [36], and *QUAS-shi* ^*ts1*^ *[36]. PBac[IT.GAL4.w+]0853* (referred to as *853-GAL4*) were identified from the InSITE collection [37] and further characterized by the first author.

The *Or22a-GAL80*, *Or85a-GAL80*, and *Or42b-GAL80* flies were generated by PCR amplifying the corresponding enhancers [6] from OregonR genomic DNA, TOPO cloning into pENTR vectors (Invitrogen), recombining with pBPGAL80Uw-6 [38] using Gateway reaction (Life Technologies), and integrating respectively into the *attP24*, *attP2*, and *attP24* sites.

### Olfactory chemotaxis assays

The four-quadrant behavioral arena was 16.5 cm by 16.5 cm, and 1 cm deep [39]. It was placed inside a 33°C box in complete darkness. The airflow was filtered and saturated with water, entered each quadrant at a rate of 40 mL/min, and left through the central hole in the arena floor. One branch of airflow was controlled by solenoid valves through the LabView software (National Instruments), so that for each trial air passed directly into one quadrant for 2.5 min, and then was switched to bubble through 5 mL of water (for vinegar and acetic acid) or paraffin oil (for all the other odorants tested) containing an odorant with a specified concentration. The odorant source was replenished for each experiment.

Flies were raised at 25°C, collected within 2–7 days of eclosion, and deprived of food for ~1 day in a vial with a wet Kimwipe. All tests were carried out in the morning peak activity window. The flies were pre-incubated at 33°C for 5 min, loaded in the arena in groups of 15–20 through a central hole in the bottom glass, tested with the aforementioned 2.5-min-air-5-min-odor paradigm, and then discarded. The “permissive temperature” control flies were raised and starved at 18°C, and then tested at 25°C. We used females throughout this study.

Illumination, tracking (30 frames/s), and pre-processing of data followed published protocols [40]. All analyses were performed with Matlab (Mathworks). The algorithm faithfully kept identities of individual flies, until they bumped into each other or walked onto the reflective edge of the arena. Each continuous segment of fly positions thus corresponded to the unambiguous trajectory of one individual. We only excluded traces that had not moved during the entirety of their extent, which corresponded to dead flies or reflections. The final dataset consisted of short trajectories, each consisting a fly’s x-y positions over consecutive frames.

**Fig 1:**
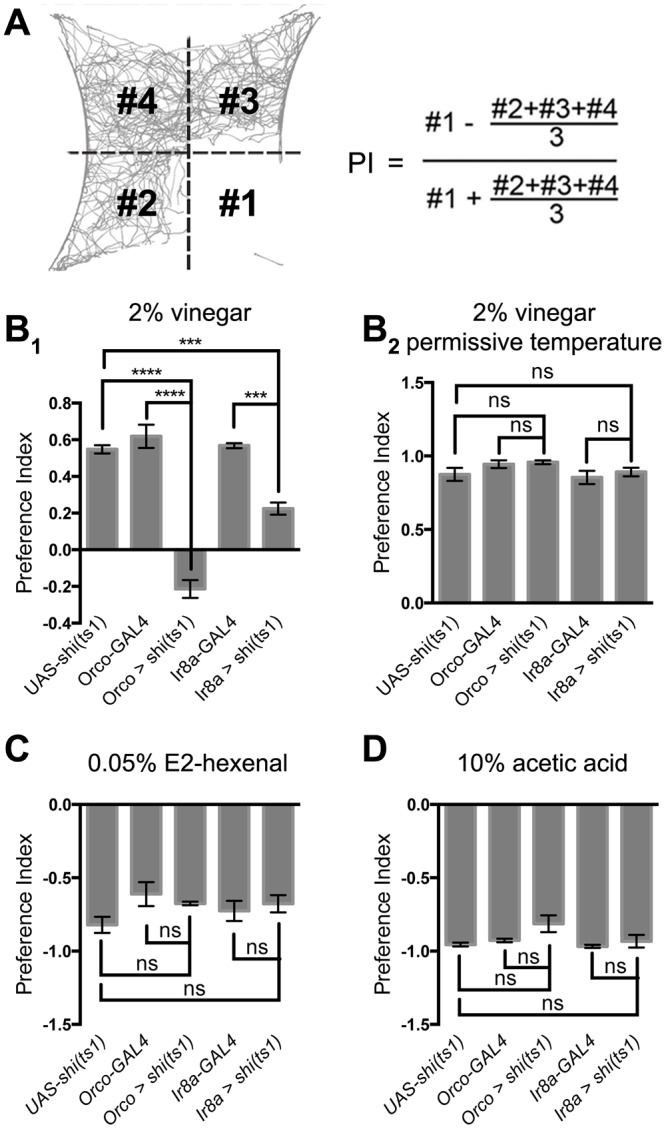
Aversion and attraction after broad ORN inactivation. **(A)** Definition of the Preference Index (PI). 10% acetic acid is delivered to the bottom right quadrant, and air to the other three quadrants. Each number (#1-#4) represents the positions visited by flies in a particular quadrant counted over all the frames in a defined period of time. **(B)** *Orco > shi* ^*ts1*^ and *Ir8a > shi* ^*ts1*^ impair attraction to vinegar at the restrictive (B_1_, n ≥ 4) but not the permissive (B_2_, n ≥ 4) temperature for *shi* ^*ts1*^. **(C, D)** The same manipulations as in B_1_ do not affect aversion to E2-hexenal (C, n ≥ 3) or acetic acid (D, n ≥ 3). Throughout this paper, n refers to the number of trials, each bar represents the mean performance index (PI), and error bars represent s.e.m.; comparisons are t-test with Holm-Bonferroni *post hoc* correction. For the same genotype on different plots, the same data were used. * p<0.05, ** p<0.01, *** p < 0.001, **** p < 0.0001.

In each frame, we then counted the number of flies in each quadrant. To calculate the preference index (PI), we added the counts from all the frames within a specific period, and then used the equation in Fig 1A. Based on a previous study [39], the PI for aversion was calculated with data between 2.5–5 min of the 5-min odorant period, during which aversion reaches steady state; the PI for attraction was calculated with data between 1–3 min, because attractive behavior appears to be desensitized after that.

### Histology

Brains were dissected from adult flies and stained using standard protocols [41]. Primary antibodies: Mouse nc82 (DSHB, 1:30) and Chicken anti-GFP (Aves Labs, 1:1000). All images were taken on a LSM 780 confocal scanning microscope with 20X or 40X objectives (Zeiss), rendered in Fiji, and assembled into figures with Photoshop (Adobe).

## Results

### Aversion is resistant to broad ORN inactivation

To quantify olfactory behavior in adult flies, we delivered innately attractive or aversive odorants to a single quadrant of an arena and air to the other three quadrants [39], and simultaneously tracked 15–20 flies in each trial (Fig 1A). We defined Preference Index (PI) as a comprehensive indicator of olfactory behavior (Fig 1A, see Methods for details), where a value of 1 means that flies never leave the odorant quadrant, a value of 0 means that flies respond neutrally to the odor and air quadrants, and a value of—1 means that the flies invariably avoid the odorant quadrant.

We first assessed the impact of ORN silencing on attraction to apple cider vinegar. ORNs can be divided into two classes expressing either an odorant receptor (OR) or an ionotropic receptor (IR). We used *Orco-GAL4* [23] to target all the OR+ ORNs, and expressed *UAS-shibire* ^*ts1*^ (*Orco > shi* ^*ts1*^), which acutely abolishes synaptic transmission at its restrictive temperature [27]. Compared to the robust attraction in control flies, blocking OR+ ORNs abolished attraction to vinegar (Fig 1B_1_); as another control, *Orco > shi* ^*ts1*^ did not affect attraction at the permissive temperature where *shi* ^*ts1*^ does not block synaptic release (Fig 1B_2_). Since acetic acid, the major component of vinegar, preferentially activates the Ir8a-expressing subset of IR+ ORNs [8], we also examined *Ir8a > shi* ^*ts1*^ flies [28], and found that they were also less attracted to vinegar (Fig 1B). Thus, normal attraction to vinegar requires both OR+ and acid-sensing IR+ ORNs.

We then tested the roles of OR+ and acid-sensing IR+ ORNs in aversion, using E2-hexenal and acetic acid, two representative chemicals preferentially activating OR+ and IR+ ORNs respectively. In contrast to attraction, aversion was not affected by blocking OR+ ORNs or acid-sensing IR+ ORNs (Fig 1C and 1D).

### Abolishing aversion with pan-ORN inactivation

**Fig 2:**
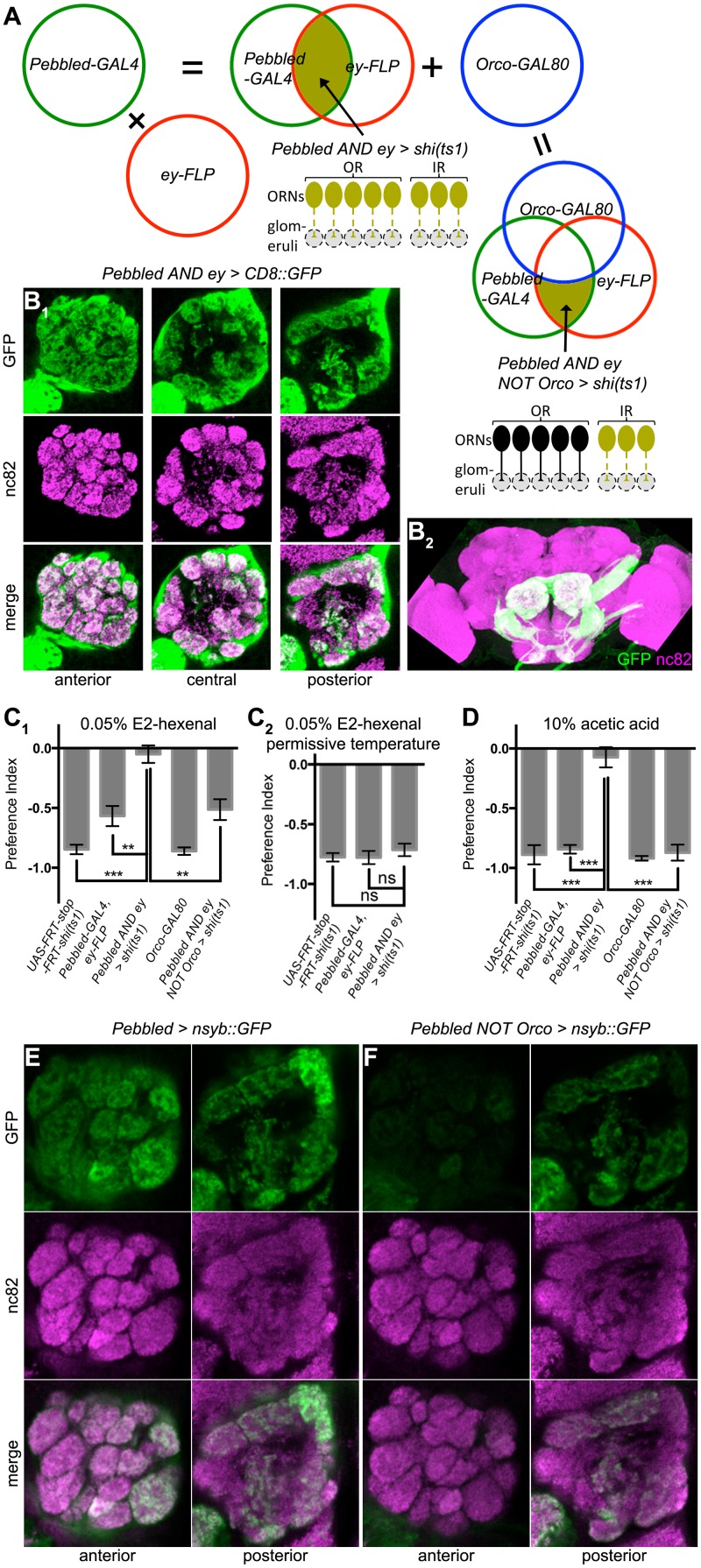
Aversion after pan-ORN inactivation and restoration in OR+ ORNs. **(A)** Venn diagrams and schematic circuits for the genetic intersections. In the Venn diagrams, green circles represent expression pattern of *Pebbled-GAL4*, red circles represent expression pattern of *ey-FLP*, blue circles represent expression pattern of *Orco-GAL80*, and dark green fill represent expression pattern of effector. In the circuit diagrams, the ellipses represent neuronal cell bodies, the straight lines represent their axon, the small triangles represent presynaptic terminals, and the grey circles in dashed lines represent glomeruli; each neuron represents one type of ORNs, black fill and solid lines indicate functional neurons, and dark green fill and dashed lines indicate blocking by the effector. Top right: AND intersection between *Pebbled-GAL4* and *ey-FLP* blocks outputs from all ORNs. Bottom left: NOT intersection by adding *Orco-GAL80* restores synaptic transmission in OR+ ORNs. **(B**_**1**_,** B**_**2**_ **)** Visualizing the intersection between *Pebbled-GAL4* and *ey-FLP* with *UAS-FRT-stop-FRT-CD8*::*GFP*. Single image slices are shown in (B_1_), and the projection of the whole brain in (B_2_). **(C)** Aversion to E2-hexenal is abolished by *Pebbled AND ey > shi* ^*ts1*^ at the restrictive (C_1_, n ≥ 3) but not the permissive (C_2_, n ≥ 4) temperature for *shi* ^*ts1*^, and rescued by *Orco-GAL80* (C_1_). **(D)** Aversion to acetic acid is also abolished by the same manipulation as in C_1_, and rescued by *Orco-GAL80* (n ≥ 4). **(E, F)** *Orco-GAL80* suppresses *Pebbled-GAL4* expression in a large subset of glomeruli, visualized with *UAS-nsyb*::*GFP*.

As aversion to acetic acid and E2-hexenal appeared resistant to the silencing of subsets of ORNs, we hypothesized that ORNs expressing different receptors redundantly mediate aversion. To test this hypothesis, we expressed *shi* ^*ts1*^ with the pan-ORN driver *Pebbled-GAL4* [29] to block synaptic transmission in all ORNs. However, *Pebbled > shi* ^*ts1*^ caused lethality, likely due to *shi* ^*ts1*^ expression outside the olfactory system. We thus used an intersectional approach to selectively target ORNs by combining *Pebbled-GAL4* with *ey-FLP* [32], a recombinase preferentially expressed in the eye-antennal disc during development [30], as well as two copies of *UAS-FRT-stop-FRT-shi* ^*ts1*^ (*Pebbled AND ey > shi* ^*ts1*^) [31]. Here the FRT-flanked stop signal is only removed by FLP in *ey+* neurons (Fig 2A). Visualized using *UAS-FRT-stop-FRT-CD8*::*GFP* [32], the intersection between *Pebbled* and *ey* specifically labeled ORNs as well as sensory neurons projecting to antennal mechanosensory and motor center and suboesophageal ganglia (Fig 2B). In the antennal lobe, every glomerulus was GFP+, indicating that we indeed targeted all ORN types (Fig 2B). As predicted, pan-ORN inactivation (*Pebbled AND ey > shi* ^*ts1*^) abolished aversion to E2-hexenal and acetic acid at the restrictive but not the permissive temperature for *shi* ^*ts1*^ (Fig 2C and 2D). For technical reasons, we could not perform the equivalent experiments for attraction, as the *Pebbled-GAL4*, *ey-FLP* control flies already showed an attraction deficit (data not shown).

To exclude the possibility that such a loss of aversion was caused by blocking the non-olfactory neurons in *Pebbled AND ey > shi* ^*ts1*^, we rescued aversion by restoring ORN outputs in the “pan-ORN inactivation” background. To do this, we introduced *Orco-GAL80* [34] to prevent *GAL4* from expressing *shi* ^*ts1*^ in OR+ ORNs (Fig 2A). To test the effectiveness of the suppression of *Pebbled-GAL4* by *Orco-GAL80*, we used a GFP tagged with neuronal synaptobrevin (nsyb) [33], which distinguishes glomeruli better than the membrane-tagged CD8::GFP. All anterior glomeruli (Fig 2E) lost GFP expression in the presence of *Orco-GAL80*, while most posterior glomeruli maintained GFP expression (Fig 2F, compare to Fig 2E), consistent with the fact that OR+ ORNs predominantly project to anterior glomeruli whereas IR+ ORNs project to posterior glomeruli [8]. *Orco-GAL80* restored aversive responses in the background of pan-ORN inactivation (*Pebbled AND ey NOT Orco > shi* ^*ts1*^) to control levels (Fig 2C and 2D).

Thus, aversion is only abolished with pan-ORN inactivation. Either OR+ ORNs (Fig 1C and 1D) or OR—ORNs (Fig 2C and 2D) are sufficient to mediate aversion at wild-type levels. This is in stark contrast to attraction, which is sensitive to the inactivation of each subset of ORNs (Fig 1B).

### Restoring outputs from specific ORNs rescues aversion

Pan-ORN inactivation offered an anosmic baseline upon which we could test the sufficiency of single ORN types in aversion. To restore synaptic transmission in ORNs expressing specific receptors, we generated GAL80 flies with the enhancers for *Or22a*, *Or42b*, and *Or85a* so that we could compare our results to a previous study [25], and we used *Ir64a-GAL80* [26] to represent IR+ ORNs.

**Fig 3:**
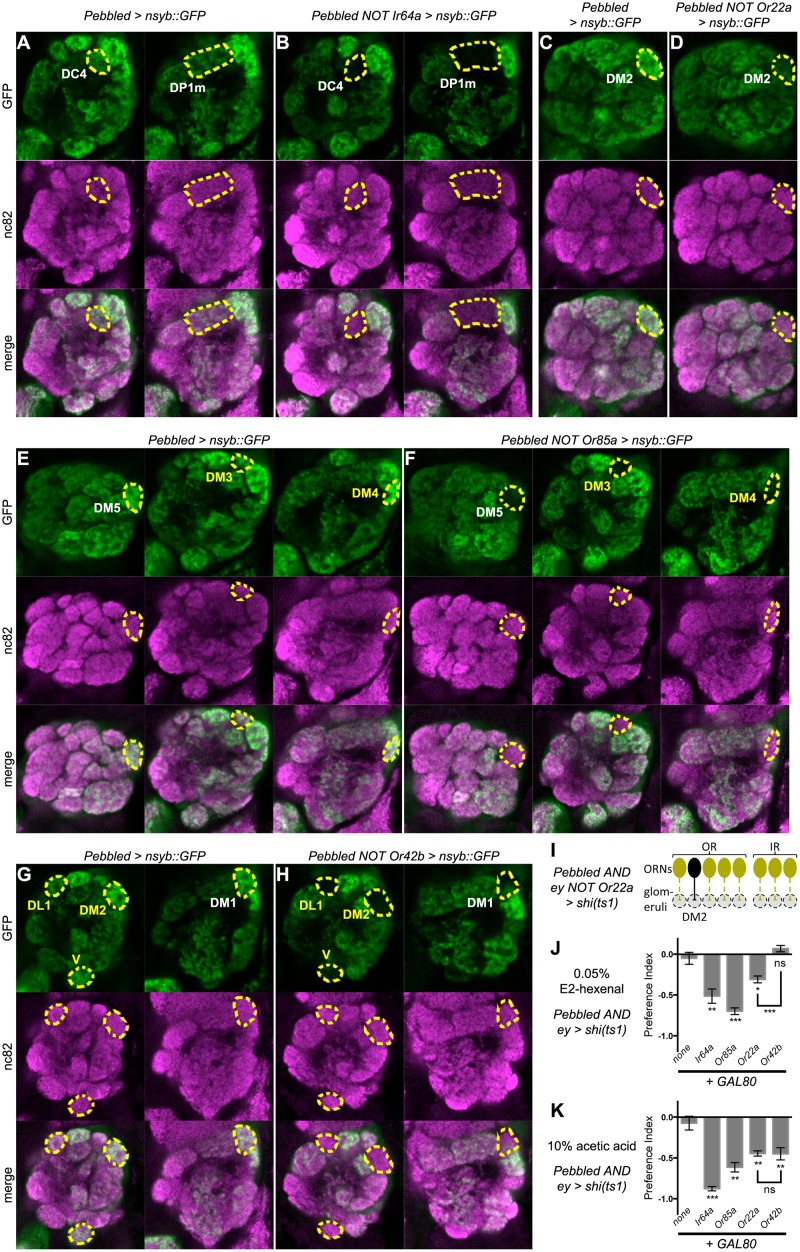
Aversion after ORN-specific restoration of synaptic transmission. **(A-H)** *Pebbled-GAL4* expression in specific glomeruli is suppressed by *Ir64a-GAL80* (A, B), *Or22a-GAL80* (C, D), *Or85a-GAL80* (E, F), and *Or42b-GAL80* (G, H). White texts label the glomeruli predicted from published work; yellow texts label the additionally suppressed glomeruli. Glomeruli with reduced expression of ORN outputs are outlined. Each image is representative of 5 brains of the same genotype. **(I)** A representative scheme of using *Or22a-GAL80* to restore inputs to DM2 glomerulus in the pan-ORN inactivation background, same legend as Fig 2A. **(J, K)** Aversion in *Pebbled AND ey > shi* ^*ts1*^ flies to E2-hexenal (J, n ≥ 3) and acetic acid (K, n ≥ 3) is recued by *GAL80s*, and *Or42b-GAL80* causes less aversion to E2-hexenal than *Or22a-GAL80* (J). The stars right below each bar indicate statistical significance as compared to the non-GAL80 control.

As with *Orco-GAL80*, we first validated the suppression of *Pebbled-GAL4* by these *GAL80* transgenes. *Ir64a-GAL80* and *Or22a-GAL80* suppressed *Pebbled* expression in the DC4+DP1m and DM2 glomeruli respectively (Fig 3A–3D), consistent with previous reports [6, 26]. In the other two cases (Fig 3E–3H), *Or85a-GAL80* suppressed DM3 and DM4 in addition to the predicted DM5, and *Or42b-GAL80* suppressed DL1, DM2, and V in addition to the predicted DM1 [6]. Thus, this strategy afforded the possibility of restoring function to a variety of ORN subsets, ranging from small groups to single types.

We next combined pan-ORN inactivation with GAL80-meditated restoration and examined aversive responses. This scheme only allows synaptic transmission in very few or single types of ORNs (Fig 3I). Despite silencing all but a few ORNs, sparse ORN outputs was sufficient to mediate aversion to various extents (Fig 3J and 3K). In the cases of *Or85a-GAL80* in E2-hexenal aversion (Fig 3J, compared to Fig 2C) and *Ir64a-GAL80* in acetic acid aversion (Fig 3K, compared to Fig 2D), PIs were restored to control levels.

Of particular interest was *Or42b-GAL80*. Since this transgene serendipitously restored activity to ORNs innervating several glomeruli including DM2 (Fig 3H), we compared its PI to that of *Or22a-GAL80*, which only targets ORN innervating DM2 (Fig 3D). *Or22a-* and *Or42b-* restoration did not differ in their aversion to acetic acid (Fig 3K), yet aversion to E2-hexenal was weaker in the latter (Fig 3J). It is possible that slightly different levels of DM2 restoration by *Or22a-* and *Or42b-GAL80s* accounts for this phenotypic difference, but it seems more likely that the additionally restored channels in the latter case signal positive valence, or at least counter the DM2-mediated aversion.

### Aversion does not require *GH146+* excitatory projection neurons (ePNs)

**Fig 4:**
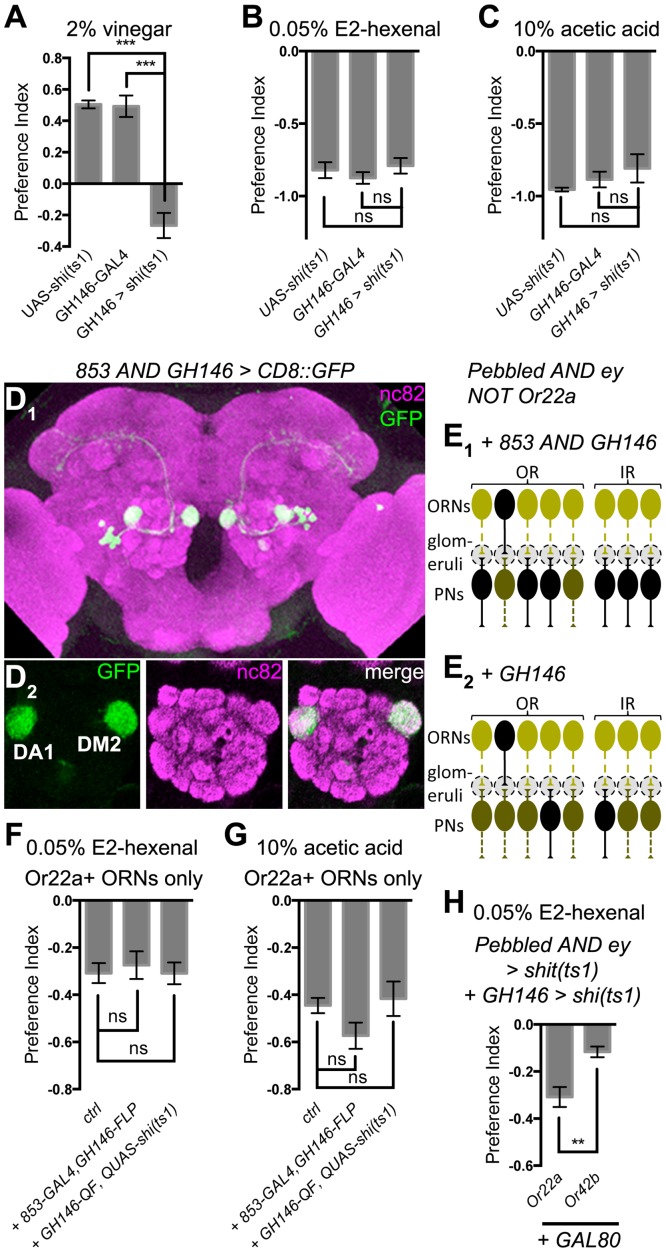
Testing the role of ePNs in aversion and attraction. **(A-C)** *GH146 > shi* ^*ts1*^ affects attraction to vinegar (A, n ≥ 4) but not aversion to E2-hexenal (B, n ≥ 3) or acetic acid (C, n ≥ 3). **(D**_**1**_,** D**_**2**_ **)** Visualizing the intersection between *853-GAL4* and *GH146-FLP* with *UAS-FRT-stop-FRT-CD8*::*GFP*. The projection of the whole brain is shown in (D_1_), and single image slices in (D_2_). **(E**_**1**_,** E**_**2**_ **)** Schemes of combining *Or22a-GAL80*-mediated restoration with the inactivation of its cognate ePNs (E_1_) or the majority of ePNs (E_2_), same legend as Fig 2A with the additional layer of ePNs connecting to cognate ORNs in the glomeruli; the color of inactivated ePNs is the intermediate between the black of intact neurons and the dark green of the inactivated ORNs, indicating possibly incomplete inactivation resulting from *GH146-GAL4* not expressing in all ePNs. **(F, G)** Aversion to E2-hexenal (F, n ≥ 4) or acetic acid (G, n ≥ 4) in *Pebbled AND ey NOT Or22a > shi* ^*ts1*^ flies (“ctrl”) is not affected by either adding *853-GAL4* and *GH146-FLP* to block the cognate DM2 ePNs, or adding *GH146-QF* and *QUAS-shi* ^*ts1*^ to block the majority of ePNs including those innervating DM2. **(H)** *Or42b-GAL80* rescue still causes less aversion than *Or22a-GAL80*, after blocking most of the ePNs with *GH146-QF* and *QUAS-shi* ^*ts1*^ (n ≥ 5).

Having tested the effects of manipulating ORN inputs to the antennal lobe on attraction and aversion, we next explored the role of antennal lobe output neurons. ePNs constitute the major output route, over 2/3 of which are labeled by *GH146-GAL4* [42]. Intriguingly, echoing the aversion/attraction dichotomy at the beginning of our study, silencing two thirds of the ePNs abolished attraction to vinegar (Fig 4A), but did not affect aversion to E2-hexenal or acetic acid (Fig 4B and 4C).

Given that a subset of ORNs were sufficient to mediate aversion, we reasoned that ePNs that do not express *GH146* might account for the robust aversion seen in *GH146 > shi* ^*ts1*^ flies. We therefore utilized aversion mediated by a single ORN type as a more stringent test for ePN functions in relaying aversive information. Each ePN receives direct input from a single glomerulus, so we first asked whether blocking output directly downstream to a single restored ORN type affects aversion. To do this, we identified an enhancer trap line [37], *853-GAL4*, which only labels ePNs innervating DM2 and DA1, in addition to expression in other brain areas (data not shown). To make this GAL4 line specific to ePNs, we intersected it with *GH146-FLP* [32] to restrict reporter expression to DM2 and DA1 ePNs (Fig 4D_1_ and 4D_2_). Surprisingly, combining the inactivation of DM2 and DA1 ePNs with the inactivation of all ORNs except those projecting to DM2 (Fig 4E_1_) did not have any effect on aversion (Fig 4F and 4G). Furthermore, aversion generated by restoring DM2 ORNs in pan-ORN inactivation was not affected even when we also blocked all *GH146+* ePNs [36], including those innervating DM2 (Fig 4E_2_, 4F, and 4G). Aversion is thus unaffected by *GH146+* ePN inactivation, even when the sensory inputs derive from only one type of ORNs. These data suggest that aversion uses antennal lobe output neurons not expressing *GH146*.

We have compared *Or22a- and Or42b-* restoration in the pan-ORN inactivation background above (Fig 3J), and concluded that the additional glomeruli rescued by *Or42b-GAL80* most likely counter DM2-mediated aversion in *Or22a-GAL80*. We tested whether such antagonism depends on *GH146+* ePNs. If *GH146+* ePNs relay positive valence from the additional glomeruli restored with *Or42b-GAL80* to higher brain centers, where the negative signal from DM2 gets canceled, blocking *GH146+* ePNs would eliminate the difference between *Or22a-* and *Or42b-* restored PIs. On the contrary, we observed that, in the *GH146* inactivation background, *Or42b-GAL80* still restored less aversion than *Or22a-GAL80* (Fig 4H). The antagonism thus does not require *GH146+* ePNs. Rather, it could take place before information leaves the antennal lobe, mediated by local interneurons; alternatively, the additional glomeruli restored with *Or42b-GAL80* could send attractive information through a yet-to-be-identified output channel (see Discussion).

## Discussion

### Aversion coding by ORNs

Previous works examined the effects of ORN-specific restoration of *Orco* in *Orco* mutants [25, 43], where inputs from IR+ ORNs were neglected. Our GAL80 restoration strategy represents the first test of the behavioral role of single ORN types by blocking all the other ORNs. Since we assessed GAL80 efficacy using suppression of GFP expression, one caveat is that we might have overlooked mild suppression by GAL80 in some glomeruli; variable strengths of different OR drivers may also subtly affect the firing of the restored ORNs. However, the predominant effect of our GAL80 strategies should be the restoration of outputs in the annotated ORNs.

**Fig 5:**
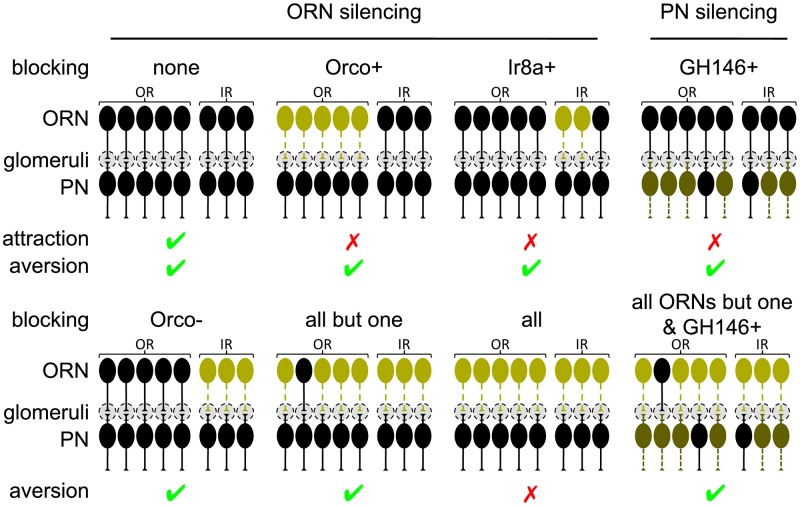
Summary of the manipulations and results. Same legend as Fig 4E. “✓” indicates the ability to perform the behavior, and “✗” indicates a behavioral defect.

With our new results (summarized in Fig 5), we can revisit the hypotheses regarding aversion coding by ORNs (see Introduction). Aversion is not only unaffected by broad ORN inactivation (Fig 1C and 1D), but also requires no more than a single ORN type in the extreme cases (Fig 3J and 3K). Hypothesis I of a “population code” for aversion is thus unlikely to be true.

The aversion restored by *Or22a-GAL80* (Fig 3J and 3K) is particularly pertinent to the rejection of “hardwired channels” in Hypothesis II. In a previous report [25], rescuing Orco expression with *Or22a-GAL4* has a neutral, if not slightly attractive, effect on olfactory behavior. In a correlative study [22], DM2 is a strong indicator of attraction rather than aversion. Taken together with our results, it thus appears that aversion can be mediated by an ORN type capable of conveying positive, neutral, or negative valence.

Although the valence conveyed by a single ORN type might depend the “context” such as the acitivty in other ORNs, it is worth noting that different ORN types might still preferentially convey aversion or attraction. *Or22a-GAL80* restores the activity of DM2 ORNs (Fig 3C and 3D), while *Or42b-GAL80* restores the activity of DM2, DM1, DL1, and V ORNs (Fig 3G and 3H), and the latter caused less aversion in response to E2-hexenal (Fig 3J). E2-hexenal does not activate DL1 ORNs [5], and V ORNs consist a well-established specialized channel for CO_2_ [3]. DM1 ORNs thus most likely accounts for the countering of DM2-mediated aversion. Moreover, in response to acetic acid, which does not activate DM1 [44], *Or42b-GAL80* did not cause less aversion than *Or22a-GAL80* (Fig 3K). These observations are consistent with the notion that DM1 ORNs are biased towards attraction [25]. We note that such effects are unlikely caused by ephaptic coupling between ORNs [45], because Or22a+ and Or42b+ ORNs are housed in different sensilla, and in general *shi* ^*ts1*^ only targets the synaptic outputs from ORNs in the antennal lobe without affecting the electric properties of ORN cell bodies in the antenna.

Given the high redundancy of aversion coding by ORNs, and the potential of coding opposite valences by a single ORN type, we favor the third scenario in introduction, where exact activity pattern within individual ORNs is more relevant than the identity or number of activated ORNs. Single types of ORNs have such coding capability, as an ORN responds to different odorants with very different dynamics [46], and it has been demonstrated that mice can use the temporal information from a single glomerulus in discriminative learning [47]. To test specific hypotheses in this class, one could perturb the temporal profile of ORN activity by manipulating receptors or ion channels and observe the change in olfactory behavior, for which our single-ORN-type strategy can be used as a paradigm.

Finally, we caution that more conditions need to be tested before our conclusions can be generalized. For example, we can’t exclude the existence of “generalist” ORN channels hardwired for attraction that we have not yet examined through either a broader odorant panel, or further utilization of our GAL80 strategy. Another caveat is that sparse ORN restoration in the background of pan-ORN inactivation might complicate interpretation: in the absence of lateral inhibition from the rest of the ORN population, the restored ORN outputs might achieve an artificially high level never possible in an intact circuit, driving an aberrant form of aversion. Finally, there is also a distinction between “innate” and “hardwired” behavior: if our pan-ORN silencing induced re-organization of the circuit in favor of aversion, we could be observing a behavior considered “innate” in the sense that it requires no training, but not “hardwired” because circuit plasticity induced by our genetic manipulation was required.

### Aversive output from antennal lobe

The lack of effect on aversion after silencing *GH146+* ePNs (Fig 4B and 4C) parallels that of broad ORN inactivation. The aversion here can be similarly interpreted as conveyed by other PNs not expressing *GH146*. Granted that typical ePNs dominate antennal lobe output in numbers, recent works suggested other neurons suitable for forwarding olfactory information to higher brain centers [48–51]. Given the functional studies mostly focused on *GH146+* ePNs, more extensive genetic manipulations of antennal lobe output neurons will be necessary to identify alternative output routes and expand our framework of understanding olfactory coding.

### The aversion/attraction dichotomy

In contrast to aversion, attraction is very sensitive to broad ORN or ePN inactivation (Fig 5). Furthermore, in our pilot experiments for this study, we could not identify a concentration of acetic acid or E2-hexenal nearly as attractive as vinegar. Both observations appear consistent with a population code for attraction. Intuitively, the identity of the odorant is coded by the activity pattern in all ORNs, and blocking half of the ORN types activated by vinegar does more than merely reducing the valence by half—the resulting input pattern is unlikely to correspond to any attractant at all. By the same token, pure chemicals are unlikely to exactly mimic the activity pattern induced by natural odorant mixtures, and are thus less attractive.

It is worth noting that, in a previous study on the execution of olfactory behaviors [39], aversion and attraction show distinct turning kinematics, and aversion-specific motor-related neurons were identified. The sensory and motor dichotomies thus suggest that, despite the apparent symmetry between aversion and attraction and their comment origin in ORN activation, they are likely relayed or deciphered by distinct downstream circuits, and processed with different logic. Future models should treat aversion and attraction as such, and should account for the observation that extremely sparse ORN inputs are sufficient for aversion to general repellents.
